# Bridging gaps in automated acute myocardial infarction detection between high-income and low-income countries

**DOI:** 10.1371/journal.pgph.0003240

**Published:** 2024-06-28

**Authors:** Nicole Chiou, Sanmi Koyejo, Christine Ngaruiya

**Affiliations:** 1 Department of Computer Science, Stanford University, Stanford, California, United States of America; 2 Department of Emergency Medicine, Stanford School of Medicine, Stanford, California, United States of America; PLOS: Public Library of Science, UNITED STATES

In the world of acute myocardial infarction (AMI)—or heart attack—care, every minute counts; the difference between life and death hinges on swift and accurate diagnosis. Now, enter the realm of artificial intelligence (AI), where groundbreaking technology is transforming the landscape of cardiac care. In Taiwan, a study unveiled a game-changing synergy: AI coupled with electrocardiogram (ECG) testing, slashing the time to diagnosis and disposition of heart attack patients by a staggering ten minutes [1]. This isn’t just about saving time; it’s about saving lives. Welcome to the future of cardiovascular medicine, where AI is not just a tool but a lifeline.

In the US, roughly 950 out of every 1,000 people who visit an emergency department (ED) with AMI concerns make it out alive [2]. This number dwindles to 596 in certain often-overlooked regions of low- and middle-income countries (LMICs) [3]. Cardiovascular disease (CVD) persists as the leading cause of mortality globally and as a major contributor to disability [4]. In 2013, the World Health Organization (WHO) set ambitious targets to reduce premature CVD mortality by 25% by 2025 and a third by 2030 [5,6]. However, in stark contrast to the decline in cardiovascular deaths witnessed in several high-income countries (HICs) [7], low-resource regions, e.g., in parts of Africa and Asia, face an alarming surge in the burden of CVD, nearing a staggering 50% increase in all-age total disability-adjusted life-years (DALYs) from over the past three decades [8]. Here, the urgent need for early detection of AMI becomes unmistakably clear, as timely medical interventions translate to reduced infarct size, preserved heart tissue, and reduced risk of post-myocardial infarction (MI) complications such as heart failure [9].

AMI diagnosis primarily relies on non-invasive ECG recording, which is typically administered within the first ten minutes of arrival to the emergency department. Currently, ECG is the most cost-effective intervention in the diagnosis and management of AMI [10]. Still, healthcare systems in LMICs grapple with constraints hindering optimal implementation of such essential diagnostics. Bottlenecked by limited access to diagnostic ECG testing, a shortage of trained personnel, and interobserver variability of interpretation, there remains a pressing need to develop innovative solutions for accurate and sustainable AMI detection. This includes redefining the landscape of AMI care for resource-limited settings and even reexamining age-old techniques—like how we “read” ECGs [11].

Amidst the challenges, the advent of automated AMI detection systems represents a paradigm shift for healthcare in LMICs. With constraints in human and financial resources, these systems emerge as invaluable clinical decision-support tools for clinicians [11]. While computer-generated interpretations have been utilized for years, they often rely on predefined rules or manual feature recognition algorithms which may not adequately capture the intricacies of an ECG [11,12]. However, the emergence of machine learning (ML) systems heralds a new era of innovation, promising to transform early detection, diagnosis accuracy, and timely intervention in AMI cases. By leveraging advanced algorithms capable of capturing complex relationships between co-occurring conditions, ML systems have the potential to identify subtle patterns indicative of AMI. Through timely diagnosis, they also enable prompt initiation of life-saving interventions, allowing resource-constrained facilities to prioritize clinical resources for severe cases–such as ST-elevation MI or larger infarcts—requiring immediate attention through advanced care or even clinical transfer. By democratizing access to advanced diagnostic tools, automated systems can revolutionize healthcare delivery and empower frontline providers.

However, while automated cardiology tools have demonstrated nascent efficacy in high-resource settings, their effectiveness remains uncertain in LMICs, where resource constraints influence their functionality and applicability [11]. Differences in AI/ML development priorities between HICs and LMICs adds to the complexity. Furthermore, the lack of heterogeneous data from LMICs presents a significant barrier to research efforts in developing and validating AI systems. Most research papers on deep learning for AMI detection are developed using the Physikalisch-Technische Bundesanstalt (PTB) database, representing the highest standard of medical care in Europe [13,14]. This database was collected at a single-site hospital in Germany, thus excluding diverse patient populations from both research development and rigorous evaluation. In addition, practical considerations in LMICs, like variations in ECG lead placement and medical equipment, further complicate system implementation and highlight the need for context-specific adaptations of automated cardiology systems research.

**Fig 1:**
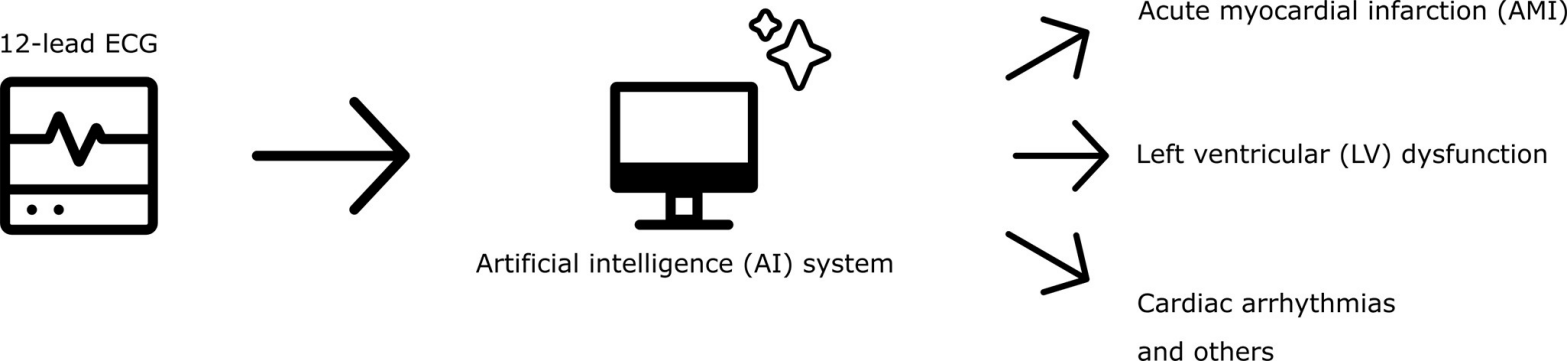
Example use cases of automated cardiology detection systems. Multiple studies have employed deep learning to detect acute myocardial infarction (AMI) and other cardiac abnormalities from 12-lead electrocardiograms (ECGs) [11,12].

While current automated solutions may not have been explicitly developed with LMICs in mind, promising research demonstrates their potential for application in resource-constrained settings. For instance, Cho et al. (2020) proposed a deep learning method for reconstructing precordial 6-lead ECGs using limb 6-lead ECGs [15], offering a cost-effective alternative when recording of the precordial leads, known for their heightened sensitivity and specificity in AMI diagnosis, are impractical or unavailable. Moreover, recent advancements in AI-enhanced electrocardiography have demonstrated the versatility of ECG-based studies in diagnostic, prognostic, and therapeutic applications for a wide range of cardiovascular diseases such as cardiac arrhythmia detection and MI localization, hinting at the vast potential of automated algorithms beyond AMI detection alone (Fig 1) [12]. These examples highlight the feasibility of automated systems in low-resource environments, informing future initiatives to enhance cardiac care in underserved communities.

All the same, while AI is a promising tool, it is no silver bullet for the complex and nuanced care that AMI demands. Yet, with so many lives at stake, dismissing its potential would be detrimental. Our focus must extend beyond the confines of algorithmic prowess, directing efforts to effectively train emergency care professionals and the fortification of comprehensive emergency care systems within which those ECGs and AMI care are housed. Addressing the continuum of care post-diagnosis is equally important, bridging the chasm between hospital interventions and care beyond the hospital, beyond the facility, and back to well-being. AI, as a tool, cannot shoulder this burden alone. Yet its potential is undeniable—streamlining diagnosis, enhancing care, influencing clinical outcomes, and ultimately, improving the quality of life [11,12]. Through its use, we can improve health and well-being, mitigating the toll of the world’s leading killer; no tool, be it stone or algorithm, should be left unturned.

A multifaceted approach is critical in charting the course for the future of automated AMI detection in low-resource settings. Firstly, research efforts should prioritize incorporating diverse and heterogeneous data sets, ensuring robust evaluation in LMICs to ascertain the applicability and efficacy of AI applications in these contexts. Secondly, in addition to improving the timing and accuracy of diagnostic tests as prioritized in HICs, technology developers must shift their focus towards creating more affordable alternatives to existing diagnostic tools and tailor solutions to address the specific challenges encountered in LMIC hospital settings. Thirdly, capacity-building on AI/ML in LMIC settings faces many of the same implementation hurdles as any new technology in health care and must enhanced to ensure readiness for its use.

Automated detection systems offer a glimmer of hope in combating the significant burden of myocardial infarction in LMICs. Through AI and machine learning, these solutions promise to revolutionize cardiac care, enabling early diagnosis and timely intervention. While successes showcase innovation’s power, realizing its full potential demands concerted efforts across research, policy, and practice. As we tackle these challenges, automated AMI detection stands as a beacon of technological advancement and a lifeline for those in the crosshairs of cardiovascular disease in the world’s most vulnerable communities.
